# Immunohistochemical expression of COX2 and iNOS in bladder cancer and its association with urinary *schistosomiasis* among Sudanese patients

**DOI:** 10.1186/1750-9378-8-9

**Published:** 2013-02-15

**Authors:** Hassan Elsiddig Hassan, Ahmed Abdel Badie Mohamed, Amel Omer Bakhiet, Hussain Gadelkarim Ahmed

**Affiliations:** 1 Department of Histopathology and Cytology, Faculty of Medical Laboratory Science Sudan University of Science and Technology, Khartoum, Sudan; 2 Department of Pathology, Faculty of Medicine, Elribat National University, Khartoum, Sudan; 3 Department of Pathology, Faculty of Vet. Medicine, Sudan University of Science and Technology, Khartoum, Sudan; 4 Department of Histopathology and Cytology, Faculty of Medical Laboratory Science, University of Khartoum, Khartoum, Sudan

**Keywords:** COX2, iNOS, Bladder cancer, *Schistosomiasis*

## Abstract

**Aims:**

The purpose of this study was to determine if any relationship exists between expression of COX2 and iNOS markers and urinary *schistosomiasis* in bladder cancers.

**Methodology:**

Immunohistochemical expression of COX2 and iNOS was assessed in formalin fixed paraffin wax processed tissues obtained from 155 patients with bladder cancers (87 SCC and 68 TCC) and 39 patients with benign bladder cystitis.

**Results:**

The overall immune-expressions of COX2 and iNOS were 71.6% and 57.2% respectively, of the 194 bladder lesions. A significant Positive association between COX2 or iNOS expression with bladder lesions (SCC, TCC and cystitis) was found (p.value = 0.000). COX2 and iNOS were co-expressed among 73(83.9%) of SCC, 15(22.1%) of TCC and 11(28.2%) of the cystitis group. The relationship between COX2 and iNOS immunostaining and Schistosomal ova positivity was statistically determined by P values 0.0565 and 0.1223 for Cox2 and iNOS, respectively.

**Conclusion:**

There are high rates of positive expression of COX2 and iNOS among Sudanese patients with Schistosomal-related bladder lesions. There might be strong association between high rates of bladder cancers and urinary Schistosomiasis in the Sudan since, the great majority of lesions were positive for COX2.

## Introduction

Bladder cancer represents a significant health problem, as it is the one of the most common cancers [1]. Worldwide, bladder cancer is diagnosed in approximately 275,000 people each year, and about 108,000 die of this disease [2].

Most investigators have accepted the association between cigarette smoking and transitional cell carcinoma (TCC), which is most prevalent in developed western and industrialized counties. In these countries over 90% of the bladder cancer cases diagnosed are transitional cell carcinomas. While in developing countries, particularly in the Middle East and Africa, the majority of bladder cancers are Squamous Cell Carcinomas (SCCs), the highest incidence has been seen in *Schistosoma* endemic areas, notably Sudan and Egypt, where SCC ranges from two thirds to three quarters of all malignant tumors of the bladder [2-4].

Cycloxgenase-2 (COX-2) is regarded as induced inflammatory mediator involved in the development of tumors [5]. It is an inducible enzyme (also called prostaglandin syntheses) responsible for conversion of arachidonic acid to prostaglandins and other inflammatory mediators [6]. It is not detectable in most normal tissues; however, it is induced at sites of inflammation by cytokines, growth factors and tumor promoters [7]. Also, prominent COX-2 expression has been described in bladder cancers including transitional cell and squamous cell carcinomas and this expression correlates with tumor grade and invasion [8,9].

Nitric oxide synthase (NOS) is the key enzyme for the conversion of L-arginine to L-citrulline and nitric oxide (NO) [10,11]. The NOS family consists of endothelial, neuronal, and inducible nitric oxide synthases (eNOS, nNOS, and iNOS, respectively) [11]. iNOS genes located on the human chromosome 17 can be induced by lipopolysaccharide, cytokines in macrophages, or tumor- related immune reactions [12,13]. iNOS was detected in human bladder cancer tissues but not in normal bladder tissues, and that it was found in macrophages and neutrophils of bladder cancer tissues and some tumor cells [14].

Bladder cancers continue to be an escalating health problem in Sudan and there is a plethora of literature incriminating urinary *Schistosomiasis* as risk factor for bladder cancer in Sudan, but confirmation for this association remains speculative. Evidences which support this association include the geographical correlation between the two conditions, and the distinctive patterns of sex and age at diagnosis. Therefore, one of the aims of this study was to elucidate the association between COX2 and iNOS expression and the *Schistosoma* related bladder lesions. Additionally, this study evaluated the application of some widely affordable conventional methods that might be useful in the early detection, management and prognostication of bladder cancer.

## Materials and methods

In this retrospective descriptive study 194 formalin-fixed, paraffin embedded bladder’s tissue blocks with their related data were retrieved from two histopathology Laboratories in Khartoum city. Out of 194 subjects, 87 were patients with bladder squamous cell carcinomas, 68 were patients with bladder transitional cell carcinomas and 39 were patients with benign bladder cystitis lesions.

### Sample processing

Serial sections on poly-L-lysine–coated slides for immunohistochemistry (IHC) and one section on a regular slide for Hematoxylin and Eosin (H&E) procedure, were prepared from each case. Sections were processed for immunostaining as follows:

The sections were de-paraffinized with xylene and then both sections were hydrated through 100%, 90%, 70% and 50% ethanol. The sections then were treated for antigen retrieved by microwave treatment for 30 minutes in citrate buffer (pH 6.0). The slides were allowed cooling for 20 minutes in the citrate buffer before further treatment. After a quick rinse in phosphate buffered saline.

Endogenous peroxidase was blocked by immersing slides in methanol with 0.3% hydrogen peroxide for 30 minutes (Dako k0411 kit).

The specimens were incubated in 5% goat serum for 10 minutes to block non-specific binding. Primary antibodies were incubated for 1 hour in a humidity chamber using the following dilutions: MIB-1 (DAKO Corp., Carpinteria, CA at a dilution of 1:50 ; COX-2 at a dilution of 1:50; polyclonal rabbit anti-iNOS antibody (Ab-1,Lab.Vision, Neo Markers, USA), Using antibody dilution at 10-20μg mL-1 and incubated for 1 hour in a humid chamber, washed in phosphate-buffered saline (PBS) incubated for 30 minutes with the secondary biotinylated antibody followed by avidin peroxidase complex for another 30 minutes according to the manufacturer's instructions (Universal Detection Kit, Dako, Denmark). A brown color was developed with 3, 3-diaminobenzidine tetra hydrochloride (DAB, Dako k0411 kit) for 5 minutes, washed in distilled water, and counterstained with Mayer's haematoxylin for 1 minute. The entire procedures were performed at room temperature. Additionally, a negative control for both markers in which the primary antibody was omitted and replaced by phosphate buffered saline was used. Positive control sections were added to process with the bladder tissue sections in the same run for precision and standardization of the elaborated IHC results.

COX2 and iNOS markers used lacked expression in normal epithelium. Therefore, normal squamous epithelium served as the control for their analysis. The immunostaining was evaluated according to the following criteria; for all three markers, when less than 5% of cells were stained positive classified as negative, less than 50% considered as low intensity, more than 50% positive for immunostaining classified as high intensity.

### Ethical consent

The study was approved by the Faculty Research Board of Sudan University for Science and Technology.

### Statistical analysis

For all statistical analyses, the SPSS statistical software version 17 was used. Pearson chi square test was used and P. values of 0.05 or less were regarded as statistically significant.

## Results

**Figure 1:**
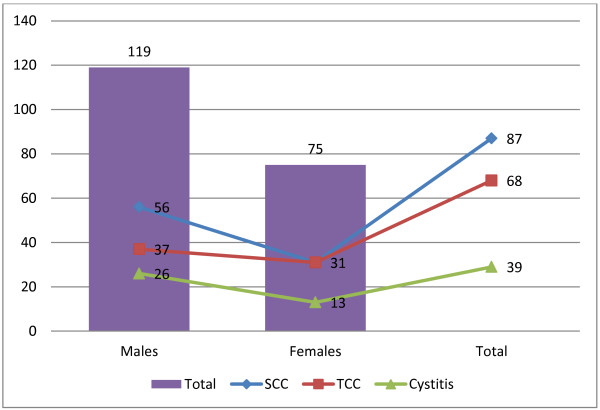
Description of the study population by Pathology and gender.

The demographic and clinic-pathological features of the involved patients were summarized in Table 1, Figure 1. The ages of the study subjects were ranging from 50 to 85 years with a mean age of 68 years. The mean age for patients with SCCs, TCCs and Cystitis were 58, 69 and 75 years, respectively. Out of 194 subjects, 119 (61.3%) were males and 75 (38.7%) were females, giving male female ratio of 1.6: 1.0. The male female rations of patients with SCCs, TCCs and Cystitis were 1.8:1.0, 1.2:1.0 and 2:1.0, respectively.

**Table 1:** The distribution of study population by age and gender

**Pathology**	**Age group**	**Gender**		**Total**	**Percent %**	**Mean ± STD**
		**Male**	**Female**			
Bladder SCC(n = 87)	≤50	15	12	27	31.0	58.06 ± 12.14
	51-60	14	09	23	26.5	
	61-70	17	04	21	24.1	
	>70	10	06	16	18.4	
Bladder TCC(n = 68)	≤ 50	03	02	05	07.4	69.49 ± 10.58
	51-60	05	04	09	13.2	
	61-70	15	06	21	30.9	
	>70	14	19	33	48.5	
Bladder cystitis(n = 39)	≤50	00	01	01	02.6	75.26 ± 10.73
	51-60	02	01	03	07.7	
	61-70	08	01	09	20.0	
	>70	16	10	26	66.7	

**Figure 2:**
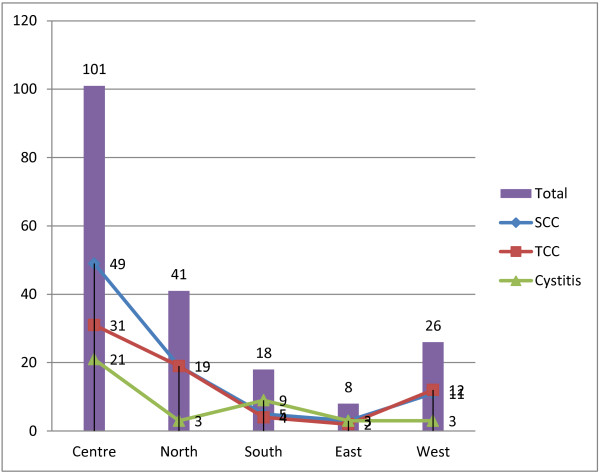
Description of the study population by Pathology and Residence.

Most patients were from Central Sudan (101/194 (52.1%)); of whom 49 (48.5%) were cases with SCCs, 31 (30.7%) were cases TCCs and 21 (20.8%) were patients with bladder cystitis, followed by Northern Sudan, Western Sudan, Southern Sudan and Eastern Sudan constituting 21.1%, 13.4%, 9.3% and 4.1%, respectively, as shown in Figure 2.

Of the 194 bladder tissue samples, Schistosoma ova was detected in 50/194 (25.8%), of which 28/50 (56%), 10/50 (20%) and 12/50 (24%) were found in SCC, TCC and cystitis respectively. COX2 positive expression was identified in 47/50 (94%) of tissues with positive Schistosoma ova (see Additional file 1 colored plate (1). However, 1 (2%) of TCC and 2 (4%) of Cystitis were found with negative COX2 expression.

iNOS positive expression was identified in 44/50 (88%) of tissues with positive Schistosoma ova (P <0.00001) of which 28/44 (63.6%), 6/44 (13.7%) and 10/44 (22.7%) were found in SCC, TCC and cystitis, respectively (Additional file 1 see colored plate (2,3). However, 4 (8%) of TCC cases were detected with negative COX2 expression.

The overall COX2 positive expression was identified in 94.3%, 57.4% and 46.2% of SCC, TCC and Cystitis, respectively, with P value = 0.0001 for SCC and P = 0.2824 for TCC. Low COX2 staining intensity was demonstrated in 63.2%, 44.1% and 46.2% of the SCC, TCC and Cystitis respectively, with P value = 0.0148 for SCC and P = 0.0234 for TCC. High COX2 staining intensity was demonstrated in 31.1%, 16.2% and 0% of the SCC, TCC and Cystitis respectively, with P value = 0.037 for SCC and P = 0.088 for TCC, as indicated in Table 2, Additional file 1 colored plate 3.

**Table 2:** The immunohistochmical of COX2 iNOS markers among bladder pathological conditions

**Variable**	**Category**	**SCC**	**TCC**	**Cystitis**
COX2 expression	Positive	82 (94.3%)	39 (57.4%)	18 (46.2%)
	Negative	5 (5.7%)	29 (42.6%)	21 (53.8%)
	P value	0.0001	0.2824	0
COX2 staining intensity	Low	55 (63.2%)	30 (44.1%)	18 (46.2%)
	High	27 (31.1%)	11 (16.2%)	0 (0%)
	P for low	0.0148	0.0234	0
	P for high	0.037	0.088	0
iNOS expression	Positive	74 (85.1%)	20 (29.4%)	17 (43.6%)
	Negative	13 (14.9%)	48 (70.6)	22 (56.4%)
	P value	0.0004	0.1327	0
iNOS staining intensity	Low	54 (62.1%)	19 (28%)	17 (43.6%)
	High	20 (23. %)	3 (4.4%)	0 (%)
	P for low	0.3100	0.2504	0
	P for high	0.0553	0.3649	0

iNOS positive expression was identified in 85.1%, 29.4% and 43.6% of SCC, TCC and Cystitis, respectively, with P value = 0.0004 for SCC and P = 0.1327 for TCC. Low iNOS staining intensity was demonstrated in 62.1%, 28% and 43.6% of the SCC, TCC and Cystitis respectively, with P value = 0.3100 for SCC and P = 0.2504 for TCC. High iNOS staining intensity was demonstrated 23%, 4.4% and 0% of the SCC, TCC and Cystitis respectively, with P value = 0.0553 for SCC and P = 0.3649 for TCC, as indicated in Table 2.

Positive co-expression of COX2 and iNOS was found in 73 (83.9%), 15 (22.1%) and 11 (28.2%) of SCC, TCC and Cystitis, respectively.

## Discussion

Bladder cancer is the most common urologic cancer [1]. Although, transitional cell cancer (TCC) accounts for >90% of all bladder cancers [15], in this study, SCC (56.1%) were found more than TCC (43.9%). Such findings were previously reported in a study from Egypt [3]. However, health administration records showing that, SCC is the commonest histopathological variant of bladder cancer in the Sudan. This is completely reverse to reports from western world (90% for TCC versus 6–8% for SCC) [16] where Schistosomiasis is not endemic and therefore, not a significant factor in the etiology of bladder cancer.

The great majority of patients in this study (52.1%) were from Central Sudan, where Schistosomiasis is endemic. Similar findings were previously reported by a study [17] investigated urinary schistosomiasis in 255 urinary bladder cancer patients from Central Sudan. The study concluded that the pattern of bladder cancer conformed to that reported from regions with endemic schistosomiasis.

The majority of studied cases were males (64.4% of the SCC cases), (54.4% of the TCC group) and (66.7 % of the cystitis group), probably because males usually performing the agricultural work in the Sudan and therefore, are more exposed to schistosomiasis-infected water.

The mean age was 58 years for bladder SCC which is consistent with reports from Schistosomiasis endemic areas; hence, the mean age for TCC was 48 years old. Such findings were previously reported [18].

Since, identification of COX-2 is useful in patient diagnosis and treatment or clinical management [19]. COX-2 was markedly expressed in the cytoplasm of most of bladder SCC (94.3%). In contrast, TCC was frequently less expressed COX-2 (57.4%). These differences in expression patterns might be attributed to differences in etiology. The development of squamous cell carcinoma is closely correlate with chronic urinary tract infection; therefore, COX-2 plays an important role in inflammation-induced carcinogenesis. These findings agree with some studies [20,21] in this context. These studies found that COX-2 is expressed in squamous cell carcinomas of the urinary bladder and in the precursor lesions. Concerning COX2 staining intensity, High COX2 staining intensity was demonstrated in SCC and TCC with P value = 0.037 for SCC and P = 0.088 for TCC. Hammam, *et al*. [22] couldn’t agree with the present results, they found that COX-2 reactivity was higher in transitional cell carcinoma (TCC) than in squamous cell carcinoma (SCC) (P < .01).

COX-2 was strongly associated with schistosomal ova detection, since all of the SCC cases that were positive for schistosoma ova were found positive for COX-2 marker. However, only 9.3% of negative schistosomal ova SCC samples were negative for COX-2. Furthermore, 90% of schistosomal ova positive TCC were found to express COX-2, while 48.3% of schistosomal ova negative TCC were negative for COX-2. These findings suggest that schistosomal inflammation stimulates production of COX-2, and the increased level of COX-2 metabolically activates nitrosamines, which are produced in patients with chronic urinary tract infection, resulting in the development of squamous cell carcinoma. Similar findings were previously reported [23] in a study revealed a significant difference in COX-2 alterations between patients with bilharzial related bladder cancer and those with non-bilharzial related bladder cancer (p < 0.05), they concluded that, their findings support the need for further evaluation of COX-2 and inflammatory signaling pathways as well as COX-2-targeted prevention and therapies in Bladder Cancer.

Nitric oxide (NO) is synthesized by the enzyme family of nitric oxide synthases (NOS) and plays an important role in tumor growth and angiogenesis. In regard to iNOS expression, it was found in 85.1% of bladder SCC, 29.4% TCC, with P value = 0.0004 for SCC and P = 0.1327 for TCC. Strong association between schistosomal ova positivity and immunoreactivity of iNOS was detected. Hundred percent of schistosomal ova positive bladder SCC were immunopositive for iNOS, and among 60% of TCC and 100% of cystitis.

NO has several diverse biological functions, and is produced by many cell types other than endothelium [24,25]. Several reports on the possible role of NOS in neoplasia have been published recently [26,27]. In a study, on mechanisms underlying development of urothelial carcinomas (UCs) of the urinary bladder associated with Schistosomiasis, the study immunohistochemically analyzed the relationship between oxidative stress markers, DNA single strand breaks (ssDNA) which could also measure the levels of base damage and apoptosis in DNA, and expression of DNA repair genes with levels of nitric oxide synthases in bladder carcinomas of Egyptian patients with or without Schistosoma hematobium infection. Marked elevation of 8-hydroxy-2'-deoxyguanosine (8-OHdG) levels was found in squamous cell carcinomas and UCs associated with Schistosomiasis when compared with non-Schistosomal carcinomas. This was accompanied by strong over expression of the DNA-repair genes, 8-oxoguanine-DNA-glycosylase and apurinic/apyrimidinic endonuclease, as well as, increased formation levels of ssDNA. Expression levels of inducible nitric oxide synthase (iNOS) which is known to be indirectly related to oxidative stress was higher in Schistosomal than in the non-Schistosomal carcinomas. However, expression of endothelial nitric oxide synthase was slightly stronger in non-Schistosomal than in the Schistosomal carcinomas. These findings suggest a strong correlation between Schistosoma haematobium infection and increased levels of oxidative stress accompanied by a continuous DNA damage and repair in UCs, all directly correlating with elevated iNOS [28].

In conclusion, there is strong association between urinary *schistosomiasis* infection and elevated risks for developing bladder cancer in Sudan. COX-2 and iNOS are important markers that can differentiate between Schistosoma related and non- schistosoma associated bladder cancers. Accordingly both COX-2 and iNOS could be denoted as markers that can differentiate between schistosomal associated and non schistosomal associated bladder tumors. The similarities between the expressions of these tumor markers suggest a link between COX2 and iNOS pathway in bladder cancer pathogenesis.

This is consistent with a potential role for COX-2 and iNOS inhibitors in the prevention and management of this disease.

## Competing interests

The authors declare that they have no competing interests.

## Authors’ contributions

HHE: Carried out the sample processing and immunohistochemical work and participated in Manuscript draft. MAAB: Carried out the sample collection and diagnosis and participated in Manuscript draft. BAO: Carried out the sample processing and participated in Manuscript draft. AHG: Carried out manuscript preparation and general consultation. All authors read and approved the final manuscript.

## Supplementary Material

Additional file 1: **Colored plate 1.** COX2 immunohistochemical staining (Peroxidase/ DAB (brown colour), counterstained with haematoxylin in: (A) TCC (NSBT) and (B) SCC (SBT), (x400). iNOS immunohistochemical staining (Peroxidase/DAB (brown colour), counterstained with haematoxylin in: (A) TCC (NSBT) and (B) SCC (SBT), (x400). iNOS immunohistochemical staining (Peroxidase/DAB (brown colour), counterstained with haematoxylin in: (A) TCC (NSBT) and (B) SCC (SBT), (x400). (DOCX 3162 kb) Click here for file
