# Supplementary material for: Immunohistochemical expression of COX2 and iNOS in bladder cancer and its association with urinary schistosomiasis among Sudanese patients
Source: Infect Agent Cancer. 2013 Feb 15;8:9. doi: 10.1186/1750-9378-8-9 (PMC3599865; doi:10.1186/1750-9378-8-9)
Supplement: Additional file 1: — Colored plate 1. COX2 immunohistochemical staining (Peroxidase/ DAB (brown colour), counterstained with haematoxylin in: (A) TCC (NSBT) and (B) SCC (SBT), (x400). iNOS immunohistochemical staining (Peroxidase/DAB (brown colour), counterstained with haematoxylin in: (A) TCC (NSBT) and (B) SCC (SBT), (x400). iNOS immunohistochemical staining (Peroxidase/DAB (brown colour), counterstained with haematoxylin in: (A) TCC (NSBT) and (B) SCC (SBT), (x400). (DOCX 3162 kb) [file 1750-9378-8-9-S1.docx]

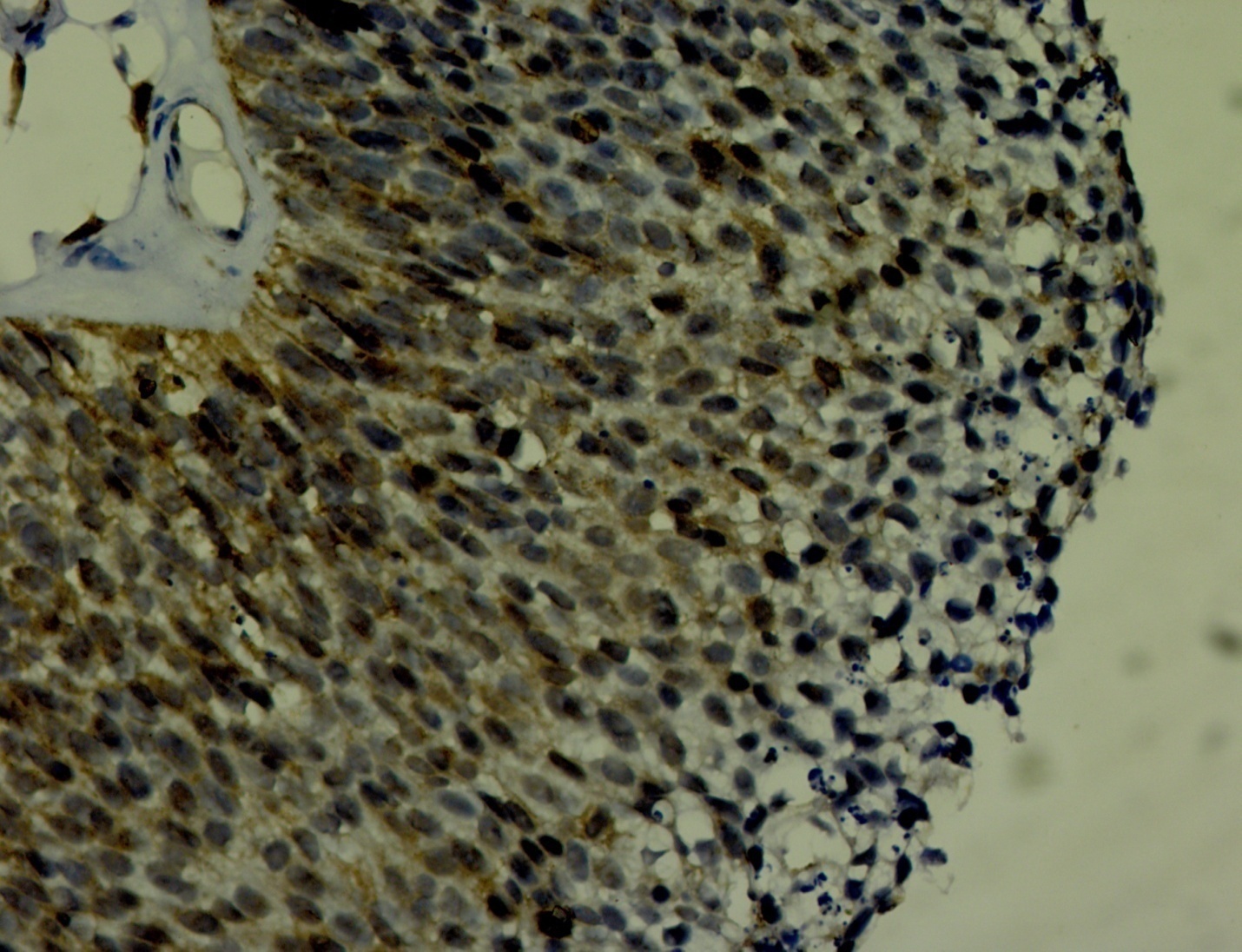


**A**


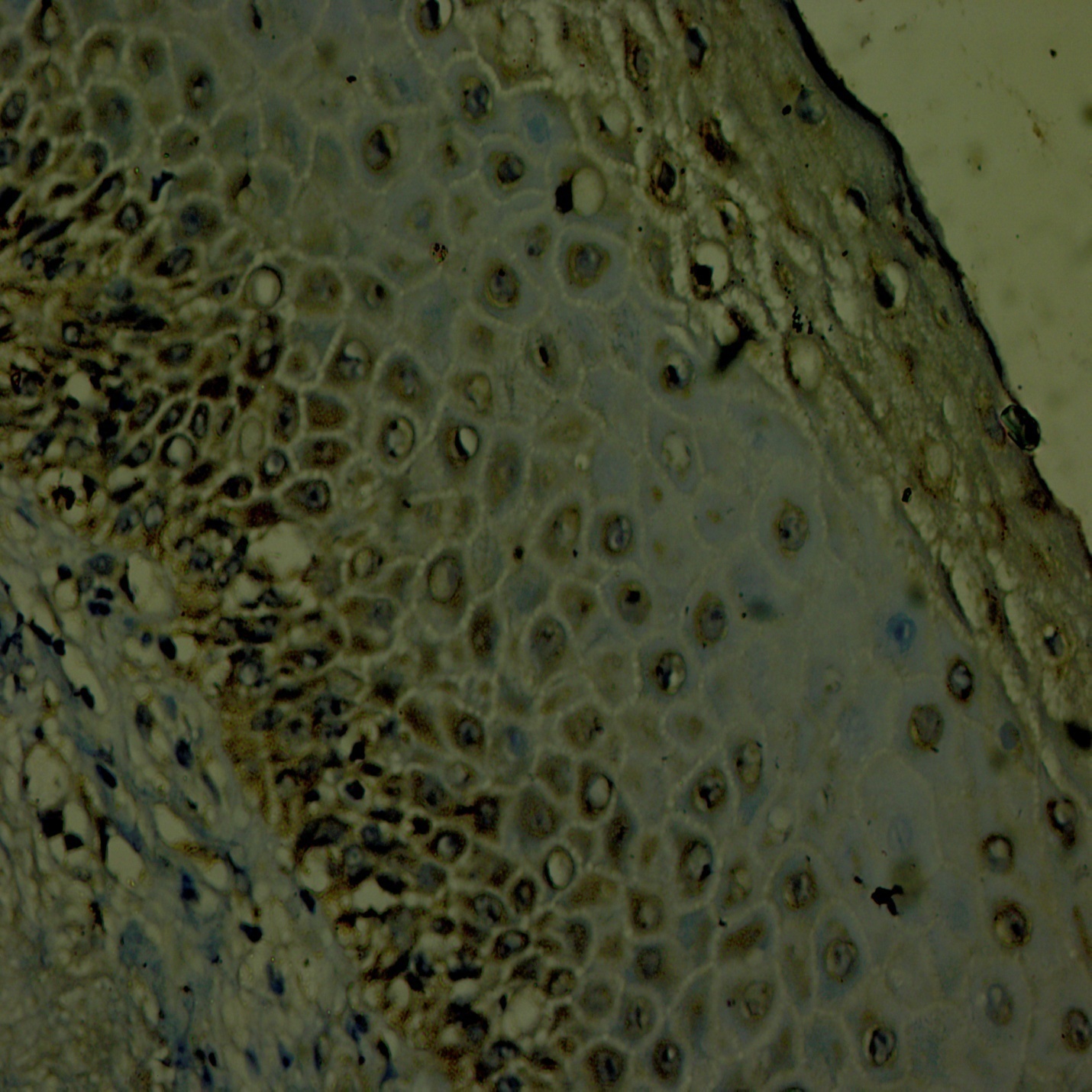


**B**

**Colored plate (1):** COX2 immunohistochemical staining (Peroxidase/ DAB (brown colour), counterstained with haematoxylin in: (A) TCC (NSBT) and (B) SCC (SBT), (x400).


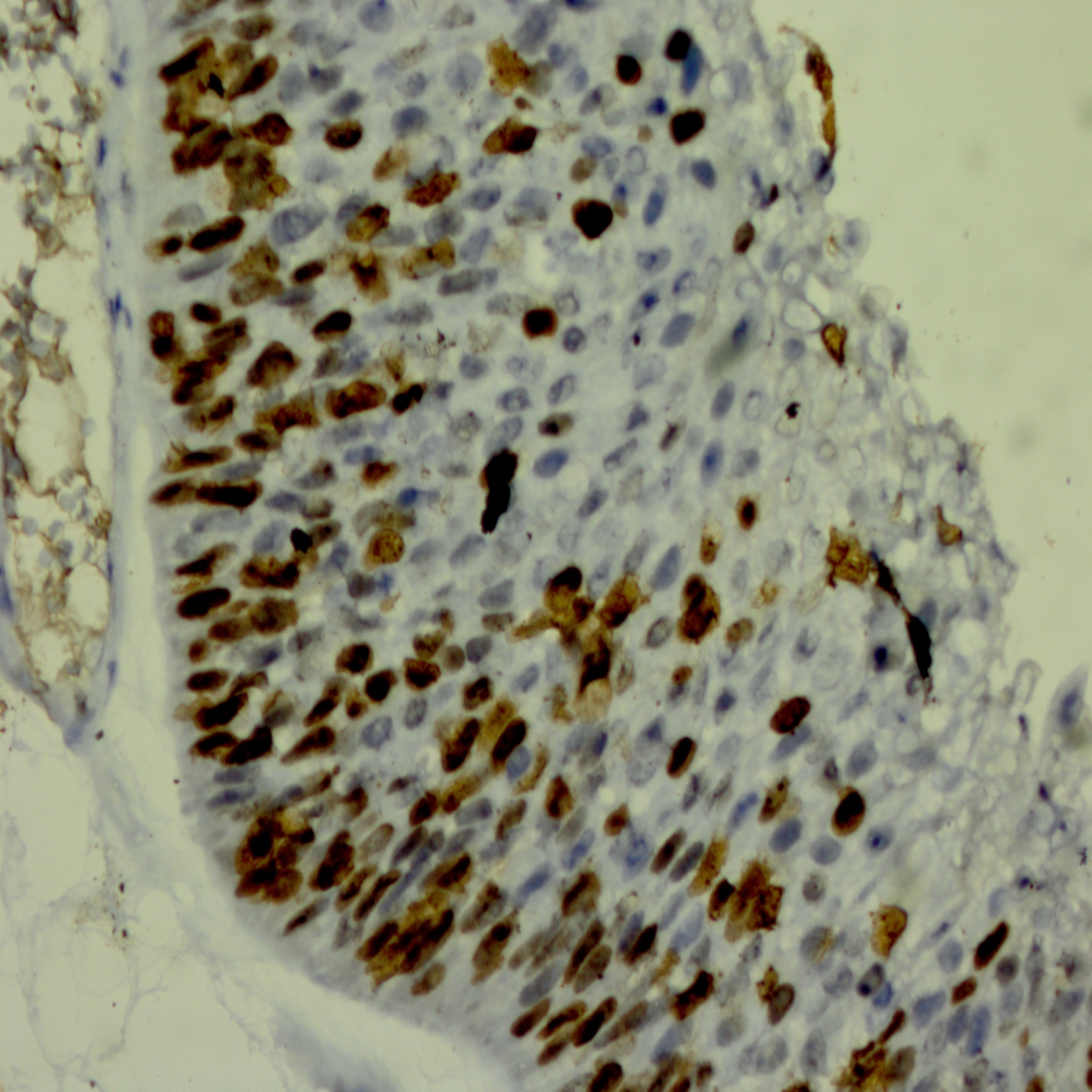


**A**

**
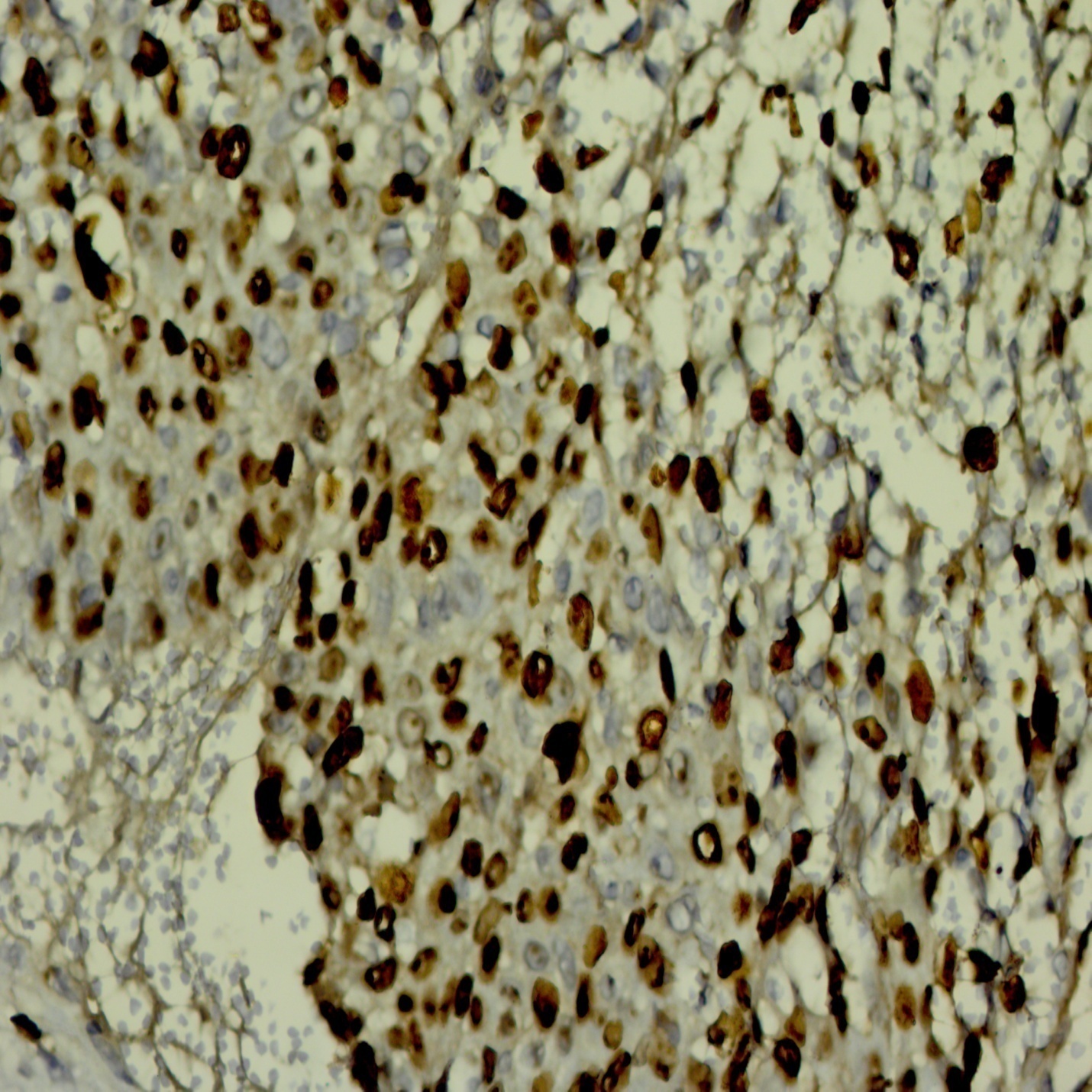
**

**B**

**Colored plate (2):** iNOS immunohistochemical staining (Peroxidase/DAB (brown colour), counterstained with haematoxylin in: (A) TCC (NSBT) and (B) SCC (SBT), (x400).


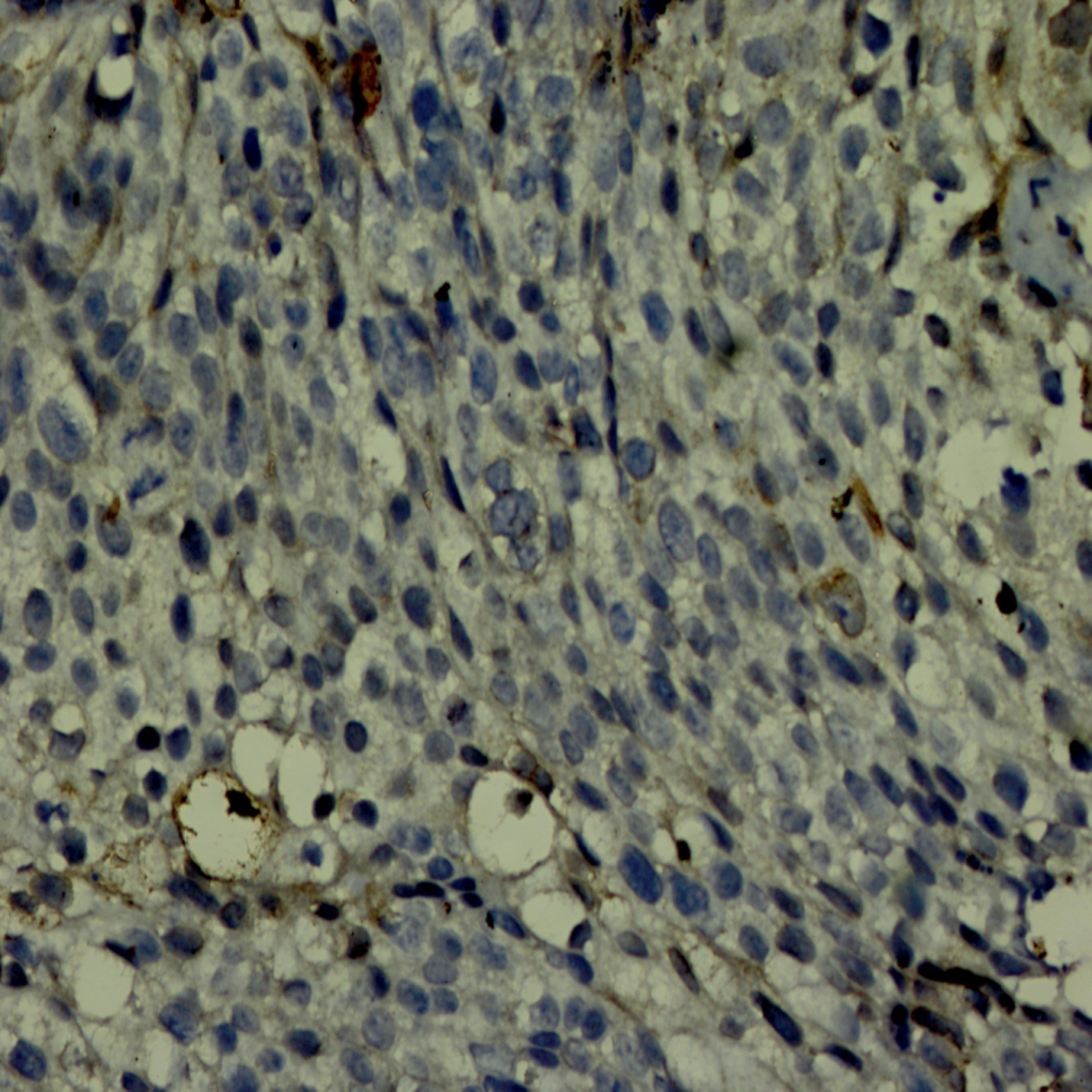


**A**


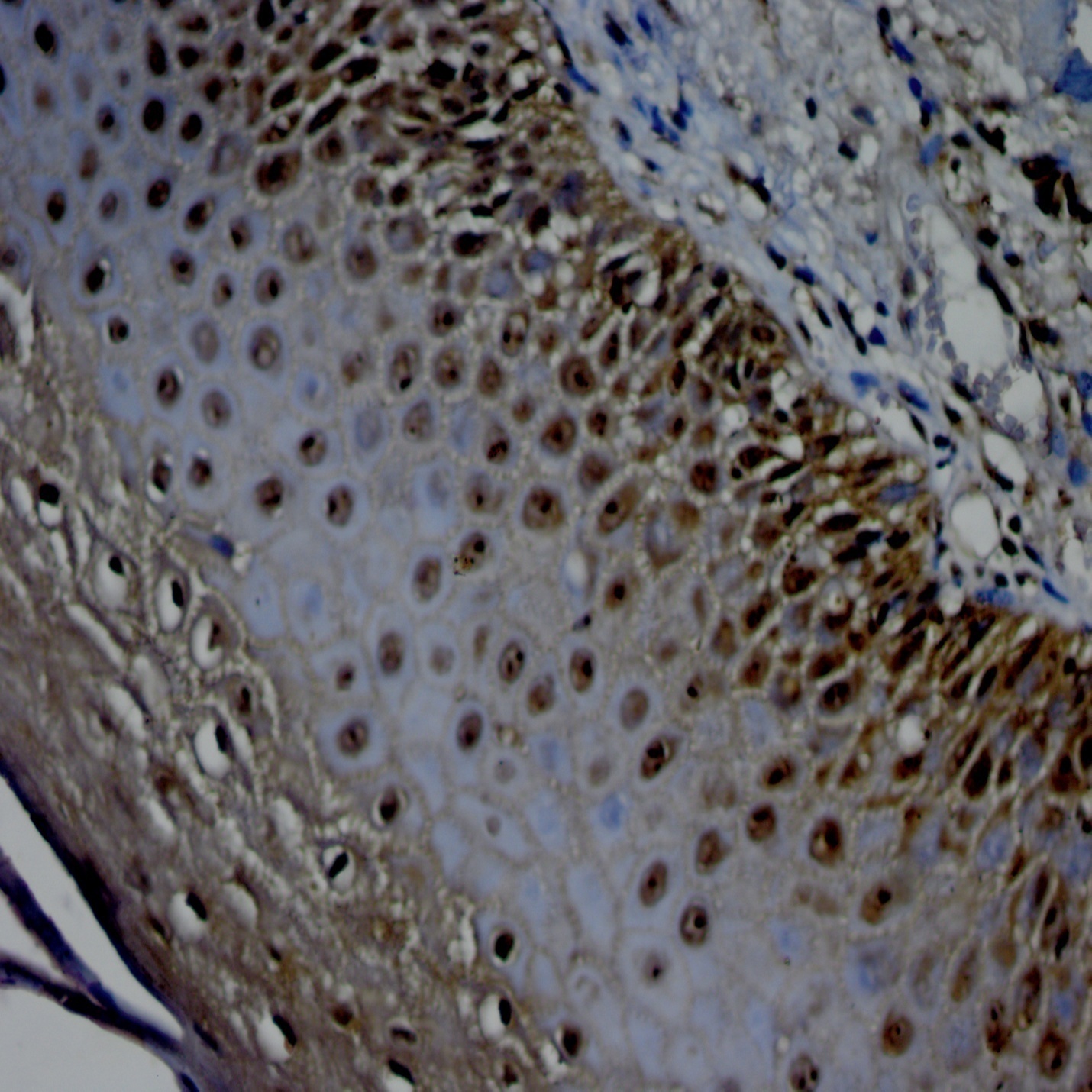


**B**

**Colored plate (3):** iNOS immunohistochemical staining (Peroxidase/DAB (brown colour), counterstained with haematoxylin in: (A) TCC (NSBT) and (B) SCC (SBT), (x400).
